# Antagonists of Growth Hormone-Releasing Hormone Inhibit the Growth of Pituitary Adenoma Cells by Hampering Oncogenic Pathways and Promoting Apoptotic Signaling

**DOI:** 10.3390/cancers13163950

**Published:** 2021-08-05

**Authors:** Iacopo Gesmundo, Giuseppina Granato, Antonio C. Fuentes-Fayos, Clara V. Alvarez, Carlos Dieguez, Maria Chiara Zatelli, Noemi Congiusta, Dana Banfi, Nunzia Prencipe, Sheila Leone, Luigi Brunetti, Justo P. Castaño, Raúl M. Luque, Renzhi Cai, Wei Sha, Ezio Ghigo, Andrew V. Schally, Riccarda Granata

**Affiliations:** 1 Division of Endocrinology, Diabetes and Metabolism, Department of Medical Science, University of Turin, 10126 Turin, Italy; iacopo.gesmundo@unito.it (I.G.); giuseppina.granato@unito.it (G.G.); noemi.congiusta@unito.it (N.C.); dana.banfi@unito.it (D.B.); nunzia.prencipe@unito.it (N.P.); ezio.ghigo@unito.it (E.G.); 2 Maimonides Institute for Biomedical Research of Córdoba (IMIBIC), Department of Cell Biology, Physiology and Immunology, University of Córdoba and Reina Sofia University Hospital, 14004 Córdoba, Spain; b22fufaa@uco.es (A.C.F.-F.); justo@uco.es (J.P.C.); bc2luhur@uco.es (R.M.L.); 3 CIBER Physiopathology of Obesity and Nutrition (CIBERobn), 28029 Madrid, Spain; 4 Centro de Investigaciones Médicas (CIMUS) e Instituto de Investigaciones Sanitarias, University of Santiago de Compostela and Complexo Hospitalario Universitario of Santiago de Compostela, 14004 Santiago de Compostela, Spain; clara.alvarez@usc.es (C.V.A.); carlos.dieguez@usc.es (C.D.); 5 Section of Endocrinology and Internal Medicine, Department of Medical Sciences, University of Ferrara, 15706 Ferrara, Italy; ztlmch@unife.it; 6 Department of Pharmacy, G. d’Annunzio University of Chieti-Pescara, 66100 Chieti, Italy; sleone@unich.it (S.L.); brunetti@unich.it (L.B.); 7 Division of Endocrinology, Diabetes and Metabolism, Department of Medicine, Miller School of Medicine, University of Miami, Miami, FL 33136, USA; renzhi.c@hotmail.com (R.C.); weisha17@gmail.com (W.S.); Andrew.Schally@va.gov (A.V.S.); 8 Endocrine, Polypeptide and Cancer Institute, Veterans Affairs Medical Center, Miami, FL 33125, USA; 9 Comprehensive Cancer Center, Department of Medicine, Miller School of Medicine, University of Miami, Miami, FL 33136, USA; 10 Division of Hematology/Oncology, Department of Medicine, Miller School of Medicine, University of Miami, Miami, FL 33136, USA; 11 Department of Pathology, Miller School of Medicine, University of Miami, Miami, FL 33136, USA

**Keywords:** GHRH, GH-secreting pituitary adenoma, ACTH-secreting pituitary adenomas, cell viability, apoptosis

## Abstract

**Simple Summary:**

Many studies have demonstrated that the antagonists of growth hormone-releasing hormone (GHRH) exert inhibitory activities in a variety of experimental cancers; however, their potential antitumor role in pituitary adenomas (PAs) remains largely unknown. Here, we show that GHRH antagonists of Miami (MIA) class, MIA-602 and MIA-690, are able to reduce the growth and promote cell death in hormone-secreting PA cell lines, through the inhibition of mechanisms implicated in tumorigenesis and cancer progression. MIA-602 and MIA-690 also decreased the viability of tumor cells derived from human pituitary tumors. Overall, these findings suggest that GHRH antagonists may represent new therapeutic tools for the treatment of PAs, both alone or in combination with standard pharmacological treatments.

**Abstract:**

Pituitary adenomas (PAs) are intracranial tumors, often associated with excessive hormonal secretion and severe comorbidities. Some patients are resistant to medical therapies; therefore, novel treatment options are needed. Antagonists of growth hormone-releasing hormone (GHRH) exert potent anticancer effects, and early GHRH antagonists were found to inhibit GHRH-induced secretion of pituitary GH in vitro and in vivo. However, the antitumor role of GHRH antagonists in PAs is largely unknown. Here, we show that the GHRH antagonists of MIAMI class, MIA-602 and MIA-690, inhibited cell viability and growth and promoted apoptosis in GH/prolactin-secreting GH3 PA cells transfected with human GHRH receptor (GH3-GHRHR), and in adrenocorticotropic hormone ACTH-secreting AtT20 PA cells. GHRH antagonists also reduced the expression of proteins involved in tumorigenesis and cancer progression, upregulated proapoptotic molecules, and lowered GHRH receptor levels. The combination of MIA-690 with temozolomide synergistically blunted the viability of GH3-GHRHR and AtT20 cells. Moreover, MIA-690 reduced both basal and GHRH-induced secretion of GH and intracellular cAMP levels. Finally, GHRH antagonists inhibited cell viability in human primary GH- and ACTH-PA cell cultures. Overall, our results suggest that GHRH antagonists, either alone or in combination with pharmacological treatments, may be considered for further development as therapy for PAs.

## 1. Introduction

Pituitary adenomas (PAs) originating from the anterior pituitary are usually benign lesions, yet they often cause comorbidities and syndromes related to local mass effects or hormonal excess. PAs account for approximately 15% of all primary intracranial tumors, with an overall estimated prevalence of almost 20% in the general population [1].

In 2017, the International Pituitary Pathology Club proposed that pituitary adenomas be termed pituitary neuroendocrine tumors (PitNETs) [2]. This topic was addressed recently at the Pituitary Neoplasm Nomenclature (PANOMEN), where it was recommended by the majority of the participants to maintain the term “adenoma” and to reconsider the topic when new evidence emerge on the biology of pituitary neoplasms [3].

PAs are heterogenous and complex, showing a multitude of proliferative and hormonal behaviors. They can either grow rapidly and promote symptoms related to intracranial mass, such as headaches, visual disorders, or hypopituitarism, or produce hormones in excess, causing severe syndromes, such as Cushing’s disease or acromegaly, thus increasing morbidity and mortality [4]. PAs can be classified as clinically non-functioning (NFPAs), and functioning (FPAs). NFPAs, which account for 14 to 54% of all adenomas, belong mainly to non-secreting tumors of gonadotroph lineage and are immunoreactive for follicle-stimulating hormone (FSH) and luteinizing hormone (LH). Functioning hypersecretory PAs include prolactin (PRL)-secreting lactotroph adenomas (PRL-PAs), which represent up to 60% of all PAs, growth hormone (GH)-secreting somatotroph adenomas (GH-PAs) (8 to 16%), adrenocorticotropic hormone (ACTH)-secreting corticotroph adenomas (ACTH-PAs) (2 to 6%), and thyroid stimulating hormone (TSH)-secreting adenomas (TSH-PAs) (less than 1%) [4,5]. Initial therapy of choice for PRL-PAs consists, usually, of dopamine agonists, while other pituitary tumors are addressed by transsphenoidal surgery and subsequent pharmacological therapy with somatostatin analogs (SSAs) and dopamine agonists, or radiotherapy for resistant or metastatic tumors [1,4,6]. The alkylating agent, temozolomide (TMZ), is also used for aggressive PRL-PAs, NFPAs, GH-Pas, or ACTH-PAs and carcinomas [6,7]. Although these drugs are usually effective in reducing hormone hypersecretion and promoting tumor shrinkage, some patients are drug-resistant and several adverse effects have been reported. Therefore, development of new drugs to treat PAs has become an urgent issue.

The hypothalamic hypophysiotropic neuropeptide growth hormone-releasing hormone (GHRH), in addition to stimulating the release of GH from pituitary somatotrophs, mediated by the pituitary type GHRH receptor (GHRH-R), exerts direct effects in extrapituitary cells and tissues by binding to both GHRH-R and its splice variant 1 (SV1) [8,9,10]. GHRH displays, among others, strong cardioprotective, anti-inflammatory and anxiolytic functions and also acts as a growth factor through autocrine/paracrine mitogenic mechanisms in both nonmalignant and tumor cells [10,11,12,13,14]. This stimulatory loop induced by GHRH can be blocked by GHRH antagonists, leading to the inhibition of tumor growth in experimental models. In fact, GHRH antagonists suppress the growth in vivo of many types of cancer, including breast, pancreatic and colorectal carcinomas, gastric and prostate cancer, lung carcinomas, malignant pleural mesotheliomas, and malignant glioblastomas [15,16,17,18,19].

Many studies demonstrated that GHRH antagonists can also strongly inhibit the stimulatory activity of exogenous and endogenous GHRH on GH secretion, suggesting that they could be used for the treatment of diseases caused by excessive production of GHRH or GH, such as acromegaly and diabetic retinopathy [20,21,22,23,24,25,26,27]. In fact, it has been demonstrated that GHRH antagonists, such as MZ-4-71 and MZ-5-156, suppressed GH and IGF-I secretion in transgenic mice overexpressing human GHRH gene, an animal model of acromegaly [20]. Furthermore, in a different study, MZ-4-71 and JV-1-36 prevented the stimulatory effect of exogenous GHRH on GH secretion in pituitary cells obtained from a human GH-secreting adenoma by blocking GHRH receptors (GHRH-Rs) [22]. The studies mentioned above were conducted with early generation antagonists, whereas the analogs of MIAMI (MIA) series, such as MIA-602 and MIA-690, synthesized more recently, possessed greatly increased anticancer activity and higher receptor binding affinity but showed only weak inhibitory activity on GH/IGF-I axis [17]. Indeed, it has been suggested that the strong inhibitory effect on tumor growth displayed by MIA antagonists could be attributed to either the downregulation of tumoral GHRH-R and SV1 levels or the conformational changes of the receptors, which would lead to different signaling responses [17,18,21,28,29].

Although the potent inhibitory effects of MIA-602 and MIA-690 have been described in many types of cancers, both in vitro and in vivo [16,18,28,29,30,31], their potential antitumor role in PAs remained to be studied. Furthermore, to the best of our knowledge, even the antineoplastic activities of earlier GHRH antagonists in PAs other than GH-PAs remains to be explored.

Based on the foregoing, in the present study, we investigated the anticancer effects of MIA-602 and MIA-690 in GH/PRL-secreting GH3-GHRHR cell line transfected with human GHRH-R, and in ACTH-producing AtT20/D16v-F2 pituitary tumor cells. Specifically, the role of GHRH antagonists was assessed on key functional parameters, such as cell viability, cell growth, apoptosis, hormone secretion, and signaling pathways. In addition, the inhibitory activity of MIA-602 and MIA-690 was determined in primary cells isolated from different human PAs, including GH-PAs, ACTH-Pas, and NFPAs.

## 2. Materials and Methods

### 2.1. Reagents

GHRH-R antagonists MIA-602 ([(PhAc-Ada)^0^-Tyr^1^, d-Arg^2^, Fpa5^6^, Ala^8^, Har^9^, Tyr(Me)^10^, His^11^, Orn^12^, Abu^15^, His^20^, Orn^21^, Nle^27^, d-Arg^28^, Har^29^]hGH-RH(1–29)NH_2_) and MIA-690 ([(PhAc-Ada)^0^-Tyr^1^, d-Arg^2^, Cpa^6^, Ala^8^, Har^9^, Fpa5^10^, His^11^, Orn^12^, Abu^15^, His^20^, Orn^21^, Nle^27^, d-Arg^28^, Har^29^]hGH-RH(1–29)NH_2_) were synthesized and purified in the laboratory of one of the authors (Andrew V. Schally) at the Veterans Affairs Medical Center, University of Miami, Miami, FL, as described previously [17]. For in vitro experiments, MIA-602 and MIA-690 were dissolved in 100% dimethyl sulfoxide (DMSO) (Sigma-Aldrich, Milan, Italy) and diluted with appropriate incubation medium. The concentration of DMSO never exceed 0.1% (vol/vol). Human GHRH(1–44)-NH_2_, rat thyrotropin-releasing hormone, mouse corticotrophin releasing hormone, temozolomide, octreotide, pasireotide, and lanreotide were obtained from Phoenix Pharmaceuticals (Karlsruhe, Germany). Dulbecco’s Modified Eagle’s medium (DMEM), 3-[4,5-dimethylthiazol-2-yl], 2,5-diphenyl tetrazoliumbromide (MTT), fetal bovine serum (FBS), bovine serum albumin (BSA), penicillin, streptomycin, amphotericin B, 3-isobutyl-1-methylxanthine (IBMX), primers for RT-PCR, and cell culture reagents were obtained from Sigma-Aldrich (Milan, Italy). Mouse monoclonal antibodies for c-Myc (sc-40), Bcl-2 (sc-7382), and actin (sc-376421) and goat polyclonal antibody for p53 (sc-1313) were obtained from Santa Cruz Biotechnology (Heidelberg, Germany). Rabbit polyclonal antibody for GHRH-R (ab76263) was obtained from Abcam (Cambridge, UK). Mouse monoclonal antibody for Bax (code: 2772) was obtained from Cell Signaling Technology (Euroclone, Milan, Italy). RT-PCR and Real-Time PCR reagents were obtained from Life Technologies, Inc. (Invitrogen, Milan, Italy).

### 2.2. Cell Culture

Rat pituitary somatolactotroph tumor cell line GH3, mouse pituitary corticotroph tumor cell line AtT-20/D16v-F2, and the C2C12 murine myoblasts cell line were obtained from the American Type Culture Collection (Manassas, VA, USA). GH3 cells expressing hGHRH-R (GH3-GHRHR) were provided by Clara V. Alvarez by co-transfection with the pCMV-hGHRH-R vector and a plasmid conferring geneticin resistance (pCMVneo) and selected with medium supplemented with 400 mg/mL G-418, as described previously [32,33]. The cells are phenotyped twice per year for expression of pituitary hormone, transcription factors, somatostatin, and GHRH receptors. Cells were maintained at 37 °C in a 5% CO_2_ humidified atmosphere in DMEM with 10% FBS, 2 mm L-glutamine, penicillin (100 U/mL), streptomycin (100 μg/mL), and 250 ng/mL amphotericin B.

### 2.3. Human Samples and Isolation of PA Cell Cultures

Human PAs samples were collected during transsphenoidal surgery (3 women with GH-PAs (mean age: 53.7 (47–51–63), 3 women with NFPAs (mean age: 60.3 (61–67–53), and 1 woman with ACTH-PAs (age: 53)). All techniques carried out in this study with human samples were conducted in accordance with the ethical standards of the Helsinki Declaration, and of the World Medical Association, and with the approval of the University of Cordoba/IMIBIC and Ethics Committees from Reina Sofia University Hospital. Informed consent from each patient was obtained. Each pituitary sample subtype was confirmed by 2 separate methods: examination by expert pathologists and by molecular screening using quantitative real-time PCR (qPCR), as previously described [34]. In all cases, fresh samples were immediately placed in sterile cold medium (S-MEM, Gibco, Madrid, Spain) supplemented with 0.1% bovine serum albumin (BSA), 0.01% L-glutamine, 1% antibiotic-antimycotic solution, and 2.5% 4-(2-hydroxyethyl)-1-piperazineethanesulfonic acid (HEPES) after surgery and dispersed into single cells and cultured, as previously reported [35]. In addition, a piece of each tumor was rapidly frozen and stored at −80 °C until extraction for total RNA and qPCR determination.

### 2.4. Cell Viability

Cells were seeded in 96-well plates at the concentration of 3 × 10^3^ cells/well. After 48 h, cells were serum starved for 12 h and further incubated with the different stimuli for a 24 h, 48 h, or 72 h. Cell viability was assessed by MTT assay, as reported previously [16]. Briefly, cells were incubated with 1 mg/mL of MTT for approximately 2 h, then the medium was removed, and formazan products solubilized with 100 μL DMSO. Cell viability was assessed by spectrophotometry at 570 nm absorbance using the LT-4000 microplate reader (Euroclone, Milan, Italy). In experiments using human primary PA cells, 1 × 10^4^ cells/well were plated in 96-well plates to assess the effect of the different stimuli on cell viability every 24 h until 72 h using Alamar blue reagent (Invitrogen, Madrid, Spain), as described previously [34,35]. Treatments were daily refreshed after each measurement, and cell viability was evaluated using Flex-Station III System (Molecular Devices, Sunnyvale, CA, USA).

### 2.5. Annexin V Analysis

Distribution of apoptotic cells was determined using the Muse Annexin V & Dead Cell Kit (Luminex, Austin, TX, USA) according to the manufacturer’s instructions. Briefly, 1 × 10^5^ cells were seeded in 60-mm dishes and, after 48 h, incubated with MIA-690 in medium supplemented with 1% FBS for 24 h and 48 h. Cells were then detached with PBS 1X/EDTA (5 mm), centrifuged (1500 rpm, 5 min), resuspended in Muse Annexin V & Dead Cell reagent, and analyzed with Muse Cell Analyzer Software (Merck Millipore, Milan, Italy) following the manufacturer’s instructions.

### 2.6. Colony Formation

To examine the influence of GHRH antagonists on colony formation, GH3-GHRHR and AtT-20 cells were seeded into 60 mm cell culture plates, at 1 × 10^3^ cells/plate, and cultured for 12 days in DMEM with 10% FBS in either presence or absence of GHRH antagonists. Then, cells were fixed with methanol, colonies were stained with crystal violet (0.05%), and plates were photographed using a digital camera (ChemiDoc XRS). Colonies were counted with Image J software (https://imagej.nih.gov).

### 2.7. Western Blot Analysis

Protein extraction and Western blot analysis were performed as described previously [16,36]. Proteins (75 μg) were resolved in 10% SDS-PAGE (12% for Bcl-2 and Bax), transferred to a nitrocellulose membrane and incubated overnight at 4 °C with the specific antibodies (dilution 1:1000 for Bax and 1:500 for GHRH-R, Bcl-2, c-Myc, and p53). Blots were re-probed with actin (dilution 1:500) for protein normalization. Immunoreactive proteins were visualized using horseradish peroxidase-conjugated goat anti-mouse, goat anti-rabbit, or mouse anti-goat (1:4000) secondary antibodies by enhanced chemiluminescence substrate (ECL) using ChemiDoc XRS (Bio-Rad, Milan, Italy). Densitometric analysis was performed with Quantity One software (Bio-Rad, Milan, Italy). Uncropped Western blot images can be found at Figures S4–S16.

### 2.8. Combination Studies

The synergistic effect produced by MIA-690 in combination with TMZ was analyzed according to the Chou-Talalay method of synergy quantitation [16,35] The Combination Index (CI) values between the two compounds were calculated using the results from cell viability assay (MTT) and calculated by CalcuSyn software. CI = 1 indicates an additive effect of the two drugs; CI > 1 indicates antagonism, whereas CI < 1 indicates synergy.

### 2.9. cAMP Assay

GH3-GHRHR cells were seeded (5 × 10^4^) in 12-well plates. After 48 h, the cells were serum-starved for 12 h and incubated in the presence of 100 μm of 3-isobutyl-1-methylxanthine (IBMX), either with or without the different stimuli. Intracellular cAMP was measured from cell lysates using the Cyclic AMP Assay (R&D System, Space Srl, Milan, Italy), according to the manufacturer’s instructions.

### 2.10. GH, PRL, and ACTH Secretion

GH3-GHRHR and AtT-20/D16v-F2 cells were seeded in 12-well plates at the concentration of 5 × 10^4^ cells/well. After 48 h, cells were serum starved for 12 h and then incubated with different stimuli at 60 min and 120 min for GH secretion and at 6 h and 24 h for PRL and ACTH secretion. Cell conditioned medium was collected and stored at −80 °C until analysis. GH, PRL, and ACTH were measured using rat GH ELISA kit (Thermo Scientific Milan, Italy), rat PRL ELISA kit (Sigma-Aldrich, Milan, Italy), and mouse ACTH EIA KIT (Phoenix Pharmaceuticals, Karlsruhe, Germany), following the manufacturer’s instructions.

### 2.11. Real-Time PCR

Total RNA isolation and reverse transcription (RT) to cDNA (1 μg RNA) from GH3 and GH3-GHRHR cells, treated with TRIzol reagent (Life Technologies, Milan, Italy), was performed as described previously [36]. Similarly, details of RNA extraction, quantification, RT, and real-time PCR of human-tissue samples using the specific primers included in this study have been reported previously [35]. For real-time PCR, reaction was performed with 50 ng cDNA, 100 nm of each primer and IQ-SYBR-green Mastermix (Bio-Rad, Milan, Italy) using the ABI-Prism 7300 (Applied Biosystems, Milan, Italy).

To control for variations in the amount of RNA used in the RT reaction and the efficiency of the RT reaction, the expression level (copy-number) of each transcript (rGHRH-R, hGHRH-R, hGHRH-Rsv1, and hGHRH) was adjusted using a normalization factor (NF) calculated from the expression of three housekeeping genes (glyceraldehyde-3-phosphate dehydrogenase (GAPDH), β-actin (ACTB), and hypoxanthine ribosyltransferase (HPRT)) in each sample by the GeNorm 3.3 Visual basic application for Microsoft Excel as previously described [37]. Results were then reported as median (minimum–maximum) of the copy number-background/NF of each transcript. Primers were designed with the Primer 3 Software (http://www.primer3.org/), and the following primer pairs were used: *rGHRH-R*, forward 5′-CGTGGCTATTGCCATCCTGGTT-3′, reverse 5′-GGCATCCTTCAAGAACACAGCAC-3′ (XM_039107108.1); *hGHRH-R*, forward 5′-TCACCATCCTGGTTGCTCTC-3′, reverse 5′-GCAGCATCCTTCAGGAACAC-3′ (NM_000823.4); hGHRH-Rsv1, forward 5′-AGAAGGAAGGCCCCATAGTG-3′, reverse 5′-GACAGCTCCTCTGAGCAACA-3′ (NM_022659.4); *hGHRH*, forward 5′-TCGCTCTTGGTTGCTCTCTC-3′, reverse 5′-GTGATCCTCACCCTCAGCAA-3′ (NM_001184731.3).

### 2.12. Statistical Analysis

Results are presented as mean ± SEM. Significance was calculated by two-tailed Student’s *t*-test or one-way ANOVA followed by Dunnet’s or Tukey’s multiple comparison post-hoc test, as appropriate, using GraphPad Prism 5.0 (GraphPad Software, San Diego, CA, USA). Significance was established for *p* < 0.05.

## 3. Results

### 3.1. GHRH-R Is Expressed in GH3-GHRHR and AtT20 Cells

**Figure 1:**
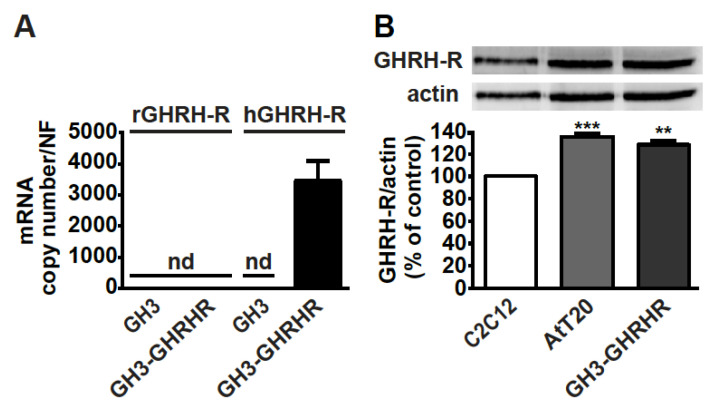
Expression of GHRH-R in GH3-GHRHR and AtT20 cells. (**A**) Real-time PCR for rat (r)GHRH-R and human (h)GHRH-R mRNAs in GH3 and GH3-GHRHR cells. Data represent absolute mRNA copy number adjusted by a normalization factor (NF), calculated from the expression levels of three control genes (GAPDH, ACTB, and HPRT) and are means ± SEM (*n* = 3) (nd, not detectable). (**B**) Representative Western blot for GHRH-R protein in AtT20 and GH3-GHRHR cells; C2C12 cells were used as positive control. Equal protein loading was determined by re-probing with antibody to actin. Results, normalized to actin and expressed as percent of C2C12 cells, are means ± SEM. ** *p* < 0.01, *** *p* < 0.001 vs. C2C12 cells by unpaired Student’s *t*-test (*n* = 3).

As GH3 cells do not constitutively express GHRH-R [38], the presence of mRNA for GHRH-R was first verified in somatolactotroph GH3 cells stably transfected with human cDNA for GHRH-R, namely GH3-GHRHR cells [33]. Real-time PCR experiments showed that, while GH3 cells were negative for both rat and human GHRH-R mRNAs, human, but not rat, GHRH-R mRNA, was present in GH3-GHRHR cells (Figure 1A). In addition, GHRH-R protein was expressed in both corticotroph AtT20 and GH3-GHRHR cells to a similar extent (Figure 1B). C2C12 myotubes were used as reference cells for GHRH-R expression, as previously reported [39].

### 3.2. MIA-602 and MIA-690 Inhibit Cell Viability and Growth of GH3-GHRHR and AtT20 Cells

**Figure 2:**
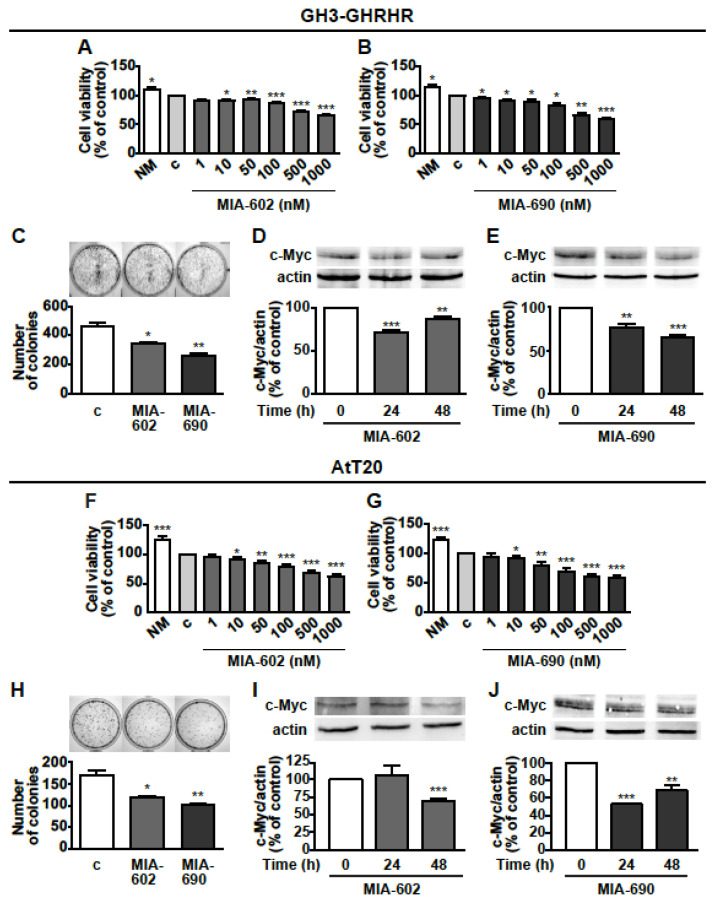
Inhibitory effects of GHRH antagonists in GH3-GHRH and AtT20 cells. Cell viability assessed by MTT in GH3-GHRH cultured in normal medium (NM) or in serum-deprived medium (c, control) for 24 h and with either MIA-602 (**A**) or MIA-690 (**B**), at the concentrations indicated. Results, expressed as percent of control are means ± SEM of three replicates. * *p* < 0.05, ** *p* < 0.01 and *** *p* < 0.001 vs. c by one-way ANOVA followed by Dunnet’s multiple comparison post-hoc test (*n* = 4). (**C**) Representative colony formation in cells treated for 12 days either with or without 1 μm MIA-602 or MIA-690. Results are means ± SEM. * *p* < 0.05, ** *p* < 0.01 vs. c by unpaired Student’s *t*-test (*n* = 3). Representative Western blot for c-Myc in cells treated with 1 μm MIA-602 (**D**) or MIA-690 (**E**) for the times indicated. Results, normalized to actin and expressed as percent of control (Time 0), are means ± SEM. ** *p* < 0.01, *** *p* < 0.001 vs. time 0, by unpaired Student’s *t*-test (*n* = 3). Cell viability assessed by MTT in AtT20 cells cultured in normal medium (NM) or in serum-deprived medium (c, control) for 24 h and with either MIA-602 (**F**) or MIA-690 (**G**), at the concentrations indicated. Results, expressed as percent of control are means ± SEM of three replicates. * *p* < 0.05, ** *p* < 0.01 and *** *p* < 0.001 vs. c by one-way ANOVA followed by Dunnet’s multiple comparison post-hoc test (*n* = 4). (**H**) Representative colony formation in cells untreated (c, control) or treated for 12 days with 1 μm MIA-602 or MIA-690. Results are means ± SEM. * *p* < 0.05, ** *p* < 0.01 vs. c by unpaired Student’s *t*-test (*n* = 3). Representative Western blot for c-Myc in cells treated with 1 μm MIA-602 (**I**) or MIA-690 (**J**) for the times indicated. Results, normalized to actin and expressed as percent of control (Time 0), are means ± SEM. * *p* < 0.05, ** *p* < 0.01 and *** *p* < 0.001 by unpaired Student’s *t*-test (*n* = 3).

Next, we investigated the potential antitumor role of GHRH antagonists in GH3-GHRHR cells. MTT assay showed that MIA-602 and MIA-690 reduced cell viability dose-dependently (1–1000 nm), and similarly after treatment for 24 h, compared with untreated cells. The reduction was 11.4%, 27.5%, and 32.5% for MIA-602 at 100, 500, and 1000 nm concentrations, respectively (Figure 2A); and 15.8%, 33.4%, and 42.2% for MIA-690 at 100, 500, and 1000 nm, respectively (Figure 2B). The most effective concentration for both peptides in terms of reduction of cell viability was 1000 nm (1 μm), which was selected for subsequent studies. MIA-602 and MIA-690 were also tested in non-transfected GH3 cells, where they showed no effect on both cell viability and proliferation (data not shown).

The inhibitory effect of GHRH antagonists was also compared with that of SSAs. In GH3-GHRHR cells, MIA-602 (Figure S1A,B) and MIA-690 (Figure S1C,D) reduced cell viability to an extent similar to that of octreotide at both 24 and 48 h, while no effect was observed for pasireotide and lanreotide alone. Moreover, the combination of MIA-602 or MIA-690 with each SSA did not produce a further decrease in cell viability, compared with the antagonists alone. To unveil the effect of GHRH antagonists on cell growth, we next performed colony formation assays. MIA-602 and MIA-690 inhibited the growth of GH3-GHRHR cells after 12 days of treatment, as demonstrated by the reduction in the number of colonies, compared with control (Figure 2C). In addition, both peptides blunted the expression of the oncoprotein c-Myc at 24 and 48 h, with the greatest inhibitory effect observed at 24 h for MIA-602 (Figure 2D) and at 48 h for MIA-690 (Figure 2E).

The antitumor activities of GHRH antagonists were subsequently examined in AtT20 cells. In keeping with the results obtained in GH3-GHRHR cells, MIA-602 and MIA-690 dose-dependently decreased cell viability and 1 μm was the most effective concentration. The reduction was 21.2%, 31.5%, and 37.1% for MIA-602 at 100, 500, and 1000 nm, respectively (Figure 2F), and 29.6%, 38.7%, and 41.8% for MIA-690, at the same concentrations (Figure 2G). The inhibitory effect of MIA-602 was greater than that of pasireotide and lanreotide at 24 and 48 h, respectively (Figure S1E,F); interestingly, MIA-690 was more effective than either pasireotide alone at 24 h, or octreotide, pasireotide and lanreotide at 48 h (Figure S1G,H). Both antagonists also similarly impaired colony formation in AtT20 cells (Figure 2H). Furthermore, c-Myc expression was downregulated at 48 h by MIA-602 (Figure 2I) and at 24 and 48 h by MIA-690 (Figure 2J). These results indicate that MIA-602 and MIA-690 inhibit cell viability and growth in both GH/PRL- and ACTH-producing PA cell lines.

### 3.3. GHRH Antagonists Promote Apoptosis in GH3-GHRHR and AtT20 Cells

**Figure 3:**
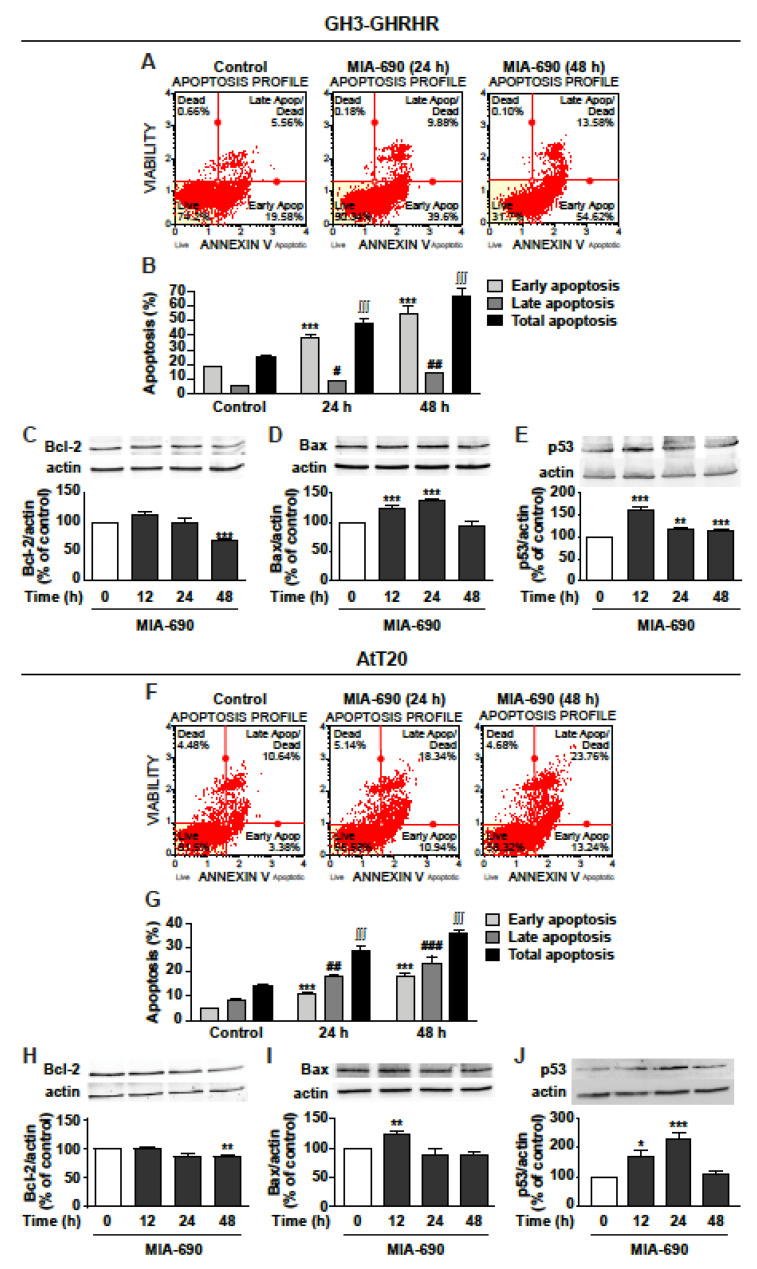
Proapoptotic effects of MIA-690 in GH3-GHRHR and AtT20 cells. (**A**) Representative results obtained by Muse Annexin V and Dead Cell assay in GH3-GHRHR cells cultured with 1% serum and treated for 24 and 48 h either without (Control) or with 1 µm MIA-690. (**B**) Quantification of the percentage of early apoptosis, late apoptosis and total apoptosis (early + late) in cells stained with Annexin V. Results are means ± SEM. *** *p* < 0.001; ^#^ *p* < 0.05, ^##^ *p* < 0.01; ^∫∫∫^ *p* < 0.001 vs. their respective controls, by unpaired Student’s *t*-test (*n* = 3). Representative Western blot for expression of Bcl-2 (**C**), Bax (**D**), and p53 (**E**) in cells treated with 1 µm MIA-690 for the times indicated. Equal protein loading was determined by re-probing with antibodies to actin. Results, normalized to actin and expressed as percent of control (Time 0), are means ± SEM. ** *p* < 0.01, *** *p* < 0.001 vs. control by unpaired Student’s *t*-test (*n* = 3). (**F**) Representative image of Annexin V staining in At-T20 cells cultured with 1% serum and treated for 24 h and 48 h without (Control) or with 1 µm MIA-690. (**G**) Quantification of early apoptosis, late apoptosis and total apoptosis (early + late) in cells stained with Annexin V. Results are means ± SEM. *** *p* < 0.001; ^##^ *p* < 0.01 ^###^ *p* < 0.001; ^∫∫∫^ *p* < 0.001 vs. their respective controls, by unpaired Student’s *t*-test (*n* = 3). Representative Western blot for Bcl-2 (**H**), Bax (**I**), and p53 (**J**) in cells treated with 1 µm MIA-690 for the times indicated. Equal protein loading was determined by re-probing with antibodies to actin. Results, normalized to actin and expressed as percent of control (Time 0), are means ± SEM. * *p* < 0.05, ** *p* < 0.01, *** *p* < 0.001 vs. control by unpaired Student’s *t*-test (*n* = 3).

Because of the similar inhibitory effects displayed by MIA-602 and MIA-690 on viability and growth of GH3-GHRHR and AtT20 cells, consistent with previous findings in other cancer cell types [16,18], the subsequent experiments were performed using only MIA-690. Analysis of annexin V staining in GH3-GHRHR showed that MIA-690 induced a strong increase in early apoptosis at 24 and 48 h, compared with control, while late apoptosis was increased to a lower extent (Figure 3A,B). Accordingly, MIA-690 reduced the expression of the antiapoptotic protein Bcl-2 at 48 h (Figure 3C) and increased the proapoptotic protein Bax, at both 24 and 48 h (Figure 3D). In addition, the tumor suppressor protein p53 was strongly increased at 12 h, and remained significantly upregulated also at 24 and 48 h (Figure 3E).

Similar results were obtained in AtT20 cells, where MIA-690 increased both early and, particularly, late apoptosis at 24 and 48 h (Figure 3F,G). Moreover, MIA-690 downregulated Bcl-2 at 48 h (Figure 3H), and elevated Bax expression at 12 h (Figure 3I), compared with control. p53 was strongly increased by MIA-690 at 12 h and, particularly, at 24 h, while decreasing to basal levels at 48 h (Figure 3J). These findings indicate that GHRH antagonists promote apoptosis and regulate the expression of critical apoptotic molecules in PA cell lines.

### 3.4. GHRH Antagonists and TMZ Synergistically Inhibit Cell Viability in PA Cell Lines

**Figure 4:**
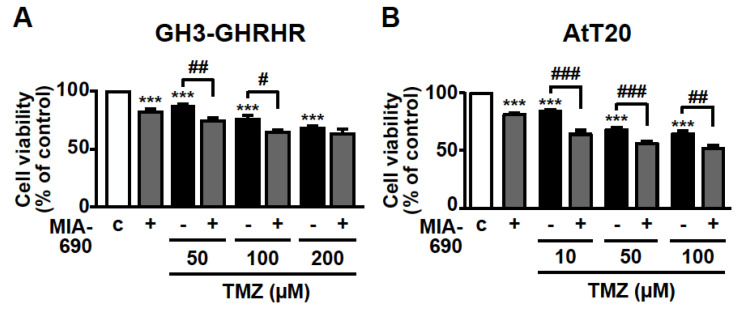
Synergistic effect of MIA-690 and TMZ in GH3-GHRHR and AtT20 cells. Cell viability, assessed by MTT, in GH3-GHRHR cells (**A**) and AtT20 cells (**B**) cultured for 72 h in medium with 2.5% serum (c, control), and with 0.1 µm MIA-690, and with or without TMZ, at the concentrations indicated. Results, expressed as percent of control, are means ± SEM of three replicates. *** *p* < 0.001 vs. c; ^#^ *p* < 0.05, ^##^ *p* < 0.01, ^###^ *p* < 0.001 by one-way ANOVA followed by Tukey’s multiple comparison post-hoc test (*n* = 4).

The inhibitory activity of MIA-690 was next examined on cell viability in combination with the chemotherapeutic agent TMZ. GH3-GHRHR cells were treated for 72 h with 0.1 μM MIA-690 and 50, 100, or 200 μm TMZ, concentrations that were selected from previous studies [40,41] and from our dose-range experiments (data not shown). TMZ alone reduced the percentage of viable cells at all the concentrations tested compared with control, particularly at 100 and 200 μm; this effect was further potentiated by MIA-690, at 50 and 100, but not 200 μm TMZ (Figure 4A). To assess the synergistic effect between TMZ and MIA-690, the MTT results were analyzed using the established method of Chou-Talalay [16,42]. The values of combination index (CI) in GH3-GHRHR cells were <0.5 (0.4 and 0.26 with 50 and 100 µm TMZ, respectively), indicating the synergism of the two compounds.

TMZ was used at lower concentrations in AtT20 cells, as these cells were more responsive to the drug compared with GH3-GHRHR cells [40,41]. In accord with the results in GH3-GHRHR cells, TMZ alone, at 10, 50, and 100 µm, dose-dependently reduced the viability of AtT20 cells at 72 h. The combination with MIA-690 further increased this effect (Figure 4B). The values of CI in AtT20 cells were <0.5 (0.22, 0.34, and 0.45 with 10, 50, and 100 µm TMZ, respectively), indicating synergism of TMZ with MIA-690.

### 3.5. GHRH Antagonists Inhibit the Expression of GHRH-R in PA Cell Lines

**Figure 5:**
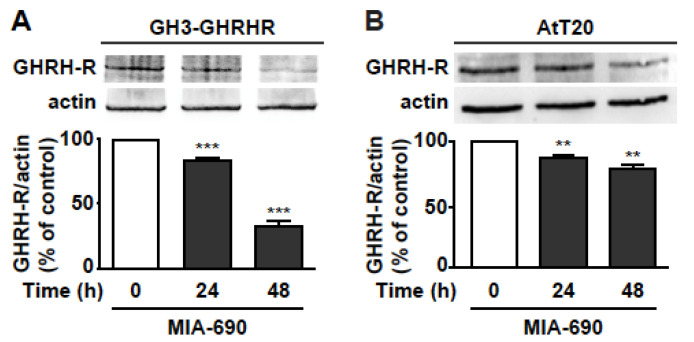
MIA-690-induced downregulation of GHRH-R in GH3-GHRHR and AtT20 cells. Representative Western blot for GHRH-R expression in GH3-GHRHR (**A**) and AtT20 cells (**B**) treated for 24 h and 48 h with 1 µm MIA-690. Equal protein loading was determined by re-probing with antibodies to actin (bottom panels). Results, normalized to actin and expressed as percent of control (Time 0), are means ± SEM. ** *p* < 0.01, *** *p* < 0.001 vs. control by unpaired Student’s *t*-test (*n* = 3).

Based on previous studies showing the ability of GHRH antagonists to reduce the expression of pituitary GHRH-R and SV1 in tumors [17,18,21,28,29], we assessed whether MIA-690 would inhibit GHRH-R levels in PA cell lines. Western blot analysis demonstrated that MIA-690 promoted a striking decrease in GHRH-R protein by 15% and 67% at 24 and 48 h, respectively, in GH3-GHRHR cells (Figure 5A), and by 13% and 22% at 24 and 48 h, respectively, in AtT20 cells, compared with cells untreated (Figure 5B).

### 3.6. MIA-690 Inhibits GH Secretion and Intracellular cAMP Levels, but Not PRL and ACTH Release in PA Cell Lines

**Figure 6:**
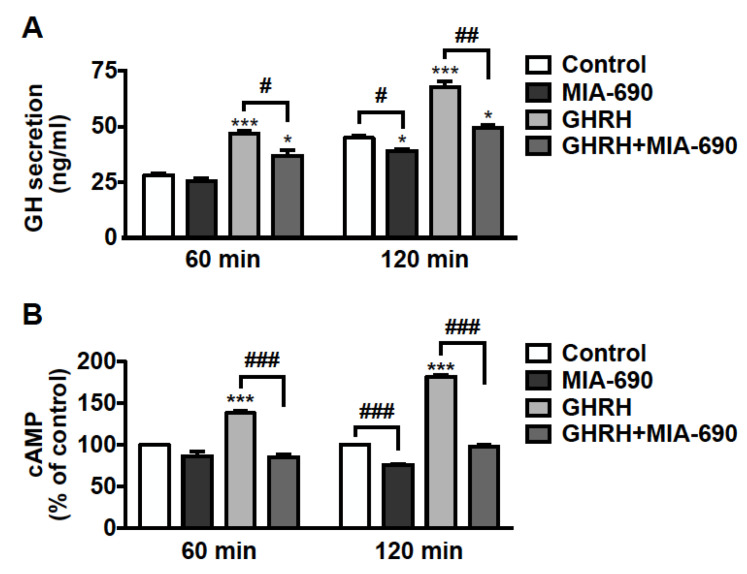
Inhibitory effect of MIA-690 on GH secretion and cAMP levels in GH3-GHRHR cells. (**A**) GH secretion assessed by ELISA in cells cultured in serum-deprived medium for the times indicated and in either absence (Control) or presence of 0.1 µm MIA-690, alone or in combination with 0.1 µm GHRH(1–44)NH_2_. Results are means ± SEM. * *p* < 0.05, *** *p* < 0.001 vs. Control; ^#^ *p* < 0.05, ^##^ *p* < 0.01 by unpaired Student’s *t*-test (*n* = 3). (**B**) Intracellular cAMP levels assessed by ELISA in cells untreated (Control) or treated with MIA-690 (0.1 µm) or GHRH (0.1 µm), either alone or in combination, for the times indicated. Results, expressed as percent of control for each time point, are means ± SEM of three independent experiments performed in triplicate. *** *p* < 0.001 vs. control, ^###^ *p* < 0.001 by unpaired Student’s *t*-test.

The effect of GHRH antagonists was further examined on hormone secretion in pituitary tumor cells. GH3-GHRHR cells were treated for 60 and 120 min with 0.1 µm MIA-690, either alone or in combination with 0.1 µm GHRH(1–44)NH_2_, to induce GH secretion. The low concentration of both MIA-690 and GHRH was established on the basis of previous studies performed in these cells with GHRH and earlier antagonists [23,43]. We found an increase in GH release after 60 min, and further increase after 120 min of treatment with GHRH. MIA-690 alone showed no effect at 60 min, while it reduced GH secretion at 120 min compared with untreated cells. Furthermore, MIA-690 counteracted the stimulatory effect of GHRH at 60 min and, particularly, at 120 min, where GH dropped almost to basal levels (Figure 6A).

We next explored the role of MIA-690 on intracellular cAMP, a critical player in GH secretion and proliferation of somatotroph cells [44,45,46,47]. GH3-GHRHR cells were exposed to 0.1 µm MIA-690, with or without 0.1 µm GHRH(1–44)NH_2_ for 60 and 120 min. MIA-690 was ineffective at 60 min but induced a decrease in cAMP levels at 120 min, compared with untreated cells. In turn, GHRH promoted a sharp rise in cAMP levels at 60 min and even more at 120 min, as expected. Notably, MIA-690 completely blocked the GHRH-induced elevation of cAMP at both time points (Figure 6B).

To examine the role of GHRH antagonists on PRL secretion, GH3-GHRHR cells were treated with MIA-690 in the presence of 0.1 µm thyrotropin-releasing hormone (TRH) [48]. While PRL levels increased after exposure to TRH, at all the time points examined, GHRH antagonists showed no effect on PRL, both alone and in combination with TRH (Supplementary Figure S2A). Similar results were obtained in AtT20 cells treated with MIA-602 and MIA-690, and with 0.1 µm corticotrophin releasing hormone (CRH) to promote the release of ACTH [49]. Indeed, whereas CRH induced ACTH secretion, GHRH antagonists did not produce any changes, both alone and with CRH (Figure S2B).

Overall, these results indicate that GHRH antagonists inhibit GH secretion in somatotroph cell lines and reduce cAMP levels, both alone and in the presence of GHRH. However, GHRH antagonists exert no effect on PRL and ACTH levels in GH3-GHRHR and AtT20 cells, respectively.

### 3.7. MIA-602 and MIA-690 Inhibit Cell Viability in Primary Human PA Cell Cultures

**Figure 7:**
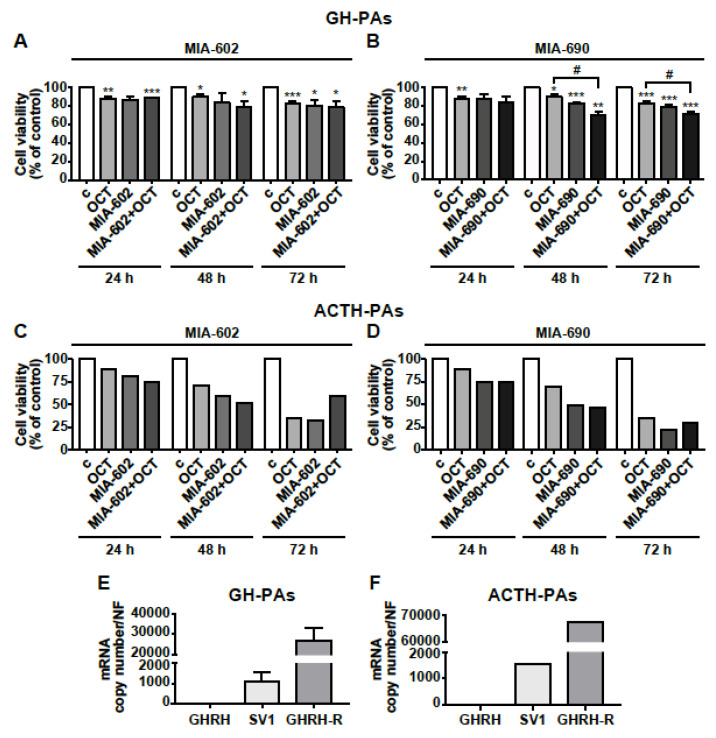
Effects of GHRH antagonists in human primary PA cell cultures. Cell viability, assessed by Alamar blue assay, in primary cells derived from GH-PAs (**A**,**B**) and ACTH-PAs (**C**,**D**). The cells were either untreated (c, control) or treated with 0.1 µm octreotide (OCT), 1 µm MIA-602 (**A**) or 1 µm MIA-690 (**B**), both alone or in combination with OCT, for the times indicated. Results, expressed as percent of control, are means ± SEM of three replicates. * *p* < 0.05, ** *p* < 0.01, *** *p* < 0.001 vs. c; ^#^ *p* < 0.05 by unpaired Student’s *t*-test *(n* = 3 for A; *n* = 2 for B; *n* = 1 for C and D). For C and D, because of *n* = 1, no error bars are presented and no statistical analysis was performed. Expression profile of GHRH, SV1, and GHRH-R in GH-PAs (**E**) and ACTH-PAs (**F**) assessed by real-time PCR. Results represent absolute mRNA copy number adjusted by a normalization factor (NF), calculated from the expression levels of three control genes (GAPDH, ACTB, and HPRT) and are means ± SEM (*n* = 3 for E; *n* = 1 for F).

The antitumor role of GHRH antagonists was finally assessed in human primary cells isolated from GH-PAs (*n* = 3), ACTH-PA (*n* = 1) and NFPAs (*n* = 3). The cells were exposed for 24, 48, or 72 h to the somatostatin analog octreotide (0.1 µm), that was used as positive control (48), or to 1 µm MIA-602 and MIA-690, either alone or in combination with octreotide. Octreotide reduced cell viability in GH-PAs at all time points tested, as expected, whereas the inhibitory effect of MIA-602 was significant only at 72 h. The combination of the two compounds showed no additional effect compared with octreotide alone (Figure 7A). Interestingly, the inhibitory effect of MIA-690 alone was greater than that of octreotide, at both 48 at 72 h; in addition, the combination of MIA-690 with octreotide produced a further inhibition, suggesting synergistic effect of the two compounds (Figure 7B).

Although it is very difficult to obtain human ACTH-PA tissues and their size is small, we had the opportunity to test the antitumor activity of GHRH antagonists in one ACTH-PA. In ACTH-PA cell culture, we observed a strong time-dependent decrease in cell viability with both MIA-602 and MIA-690 alone, an effect similar to or even greater than that displayed by octreotide. No additional effect was found for octreotide administered with MIA-602 nor MIA-690 (Figure 7C,D). In NFPA-derived cells, both the antagonists and octreotide, alone or in combination, had no effect at all the time points studied, but octreotide inhibited cell viability at 48 h only (Figure S3). Real-time PCR analysis showed that mRNA for GHRH-R was highly expressed in both GH-PA cells and, particularly, in ACTH-PA cells, while GHRH was undetectable. Furthermore, the levels of mRNA for GHRH-R were more than 26,000- and 65,000-fold higher, respectively, than SV1 in cells derived from GH-PAs (Figure 7E) and ACTH-PAs (Figure 7F), suggesting the involvement of GHRH-R, rather than SV1, in the inhibitory effect of GHRH antagonists. Interestingly, NFPA cells showed levels of GHRH, SV1 and even GHRH-R mRNAs much lower than those observed in GH- or ACTH-PA cells (Figure S3).

## 4. Discussion

In the present study, we show that MIA-602 and MIA-690, of the recent MIAMI class of GHRH antagonist, display antitumor activity in somatotroph and corticotroph PA cell lines, as well as in human primary PA cell cultures. Indeed, MIA-602 and MIA-690 possess strong inhibitory activities in various cancers [16,17,28,30,50]; however, their role in pituitary tumors still has to be investigated.

Various studies have demonstrated the anticancer effect of early generation GHRH antagonists in rat and mouse pituitary GHRH-R and human tumoral GHRH-R [20,22,23,24,25,26,27]. Furthermore, the role of GHRH antagonists of JV series in blocking GHRH actions was described in the same in vitro model of somatotroph adenoma used in this study, i.e., rat pituitary GH3 cells that were induced to express a functional human GHRH-R [23,33,43]. Indeed, because human GH-secreting PA cell lines are not available, rat GH3 cells have been widely established as a valuable alternative model. Our findings on the inhibitory effects of MIA-602 and MIA-690 in GH3-GHRHR could be partly explained by the fact that GHRH-R expression in these cells may constitutively activate or enhance, particularly upon GHRH stimulation, survival, and proliferative signaling pathways, which can be blocked by the antagonists, similarly to what occurs in AtT20 cells and GH-PA-derived cells, which express GHRH-R. Likewise, these results suggest that, despite lacking intrinsic GHRH-R, GH3 cells likely possess the signaling machinery required to convey the response to GHRH-R ligands. Actually, previous studies revealed that GH3-GHRHR are more responsive to the stimulatory effects of GHRH, on GH secretion, cAMP signaling, and proliferative pathways [23,32,33].

However, at variance with the previous findings, that were mainly focused on inhibition of GH secretion, we are the first to show the in vitro ability of GHRH antagonists to reduce cell viability and growth of PA cells, promoting apoptosis and regulating key signaling pathways involved in pituitary tumor development and progression. In fact, MIA-602 and MIA-690 similarly and dose-dependently decreased cell viability and growth of GH3-GHRHR cells, at both 24 and 48 h. Interestingly, although they were effective also at low doses, 1 µm concentration yielded the best inhibitory effect, in line with previous findings from our and other groups in different cancer cell models [16,17,18,28]. The reduction in cell viability induced by the antagonists was similar to that of octreotide, which was previously reported to be effective in these cells [51], whereas pasireotide and lanreotide alone were inactive, likely because our experimental conditions were different from those reported in other studies [52,53]. Moreover, no additional inhibition was observed when GHRH antagonists were combined with SSAs.

Of relevant importance are our findings on the antitumor activities of MIA-602 and MIA-690 in ACTH-secreting AtT20 cells, where GHRH antagonists revealed a greater ability in reducing cell viability and growth compared with octreotide, pasireotide and lanreotide. Indeed, to the best of our knowledge, the effects of GHRH antagonists of both early and latest series have never been examined in ACTH-secreting cell lines or primary cells. In addition, this is also the first study showing the presence of GHRH-R in human ACTH-PAs. It has been previously shown that human ACTH-PAs do not express GHRH-R, although only mRNA was examined and from only two tissues [54]. Overall, our findings suggest that the antitumor effects of MIA-602 and MIA-690 are not restricted to GH-secreting cells, at least with regards to the inhibition of cell viability and growth, but are also extended to other PA subtypes. Therefore, it will be intriguing, in future studies, to gain a greater understanding of the antitumor role of GHRH antagonists in pituitary tumors different from GH-PAs.

Dysregulated expression of the oncoproteins c-Myc and Bcl-2 plays an important role in tumorigenesis [55]; in addition, previous studies have demonstrated abnormal expression of c-Myc and Bcl-2 in PAs, contributing to the development and progression of these tumors [56]. Here, we show that GHRH antagonists blunted the expression of c-Myc and Bcl-2 in both GH- and ACTH-secreting cells. In addition, the tumor suppressor protein p53 and its target gene, the proapoptotic protein Bax, were upregulated in response to the antagonists. These findings are consistent with previous studies showing the involvement of these molecules in the anticancer and anti-inflammatory activities of the antagonists [16,19,57]. Interestingly, recent findings showed that DNA damage and senescence in pituitary cells in vitro, and in pituitary gland in vivo, induce the activation of the p53/p21 pathway and the increase in GH transcription and secretion, indicating that GH is a direct transcriptional target for p53. In fact, the pituitary gland has low regenerative capacity, therefore, senescence was proposed as a good alternative to apoptosis for pituitary tumor suppression [58]. In the present study, in GH3-GHRHR cells treated with GHRH antagonists, we observed an increase in p53 protein expression but not in GH secretion, suggesting induction of apoptosis rather than senescence, as supported also by the increase in annexin staining and regulation of apoptotic molecules. However, old GHRH antagonists of MZ series were previously found to increase telomerase activity and aging in mice [59]; thus, it could be worth assessing the role of MIA antagonists on senescence of PA cells in future studies.

TMZ is an alkylating chemotherapeutic drug currently used for aggressive PAs, including pituitary carcinomas; however, a large number of invasive PAs fail to respond to TMZ [6,7]. Therefore, there is a need of new therapeutic compounds to be used as an alternative or in combination with TMZ for treatment of these tumors. Here, we show that MIA-690, in addition to reducing cell viability per se, potentiated the cytotoxic effect of TMZ in both GH3-GHRHR and AtT20 cells, in a synergistic manner. This effect was even more evident in AtT20 cells, that were also more responsive to lower concentrations of the drug. In fact, TMZ has been successfully used in combination with capecitabine in aggressive corticotroph tumors [60], although studies are still required to determine the efficacy and safety of combination therapies. Overall, our findings suggest that GHRH antagonists may increase the efficacy of TMZ and allow the use of lower doses of the drug to reduce side effects.

Different studies have suggested that the downregulation and/or blockade of GHRH-R or SV1 play a major role in the antitumor activities of GHRH antagonists in vitro and in vivo [17,18,21,28,29,31]. Importantly, we found that MIA-690 promoted either a striking or moderate reduction in the expression of GHRH-R in GH3-GHRHR and AtT20 cells, respectively. Collectively, these findings suggest a direct inhibition of survival/proliferative pathways in both cell lines mediated by GHRH-R, and possible blockade of the autocrine/paracrine stimulatory action of GH in GH3-GHRHR cells, as reported for other tumors [21]. Some studies have previously proposed that the antitumor effects of GHRH antagonists could be also due to conformational changes of GHRH-Rs, which would induce distinct mechanisms and regulatory responses [17,61]. Noteworthy, it has been recently shown that the agonistic GHRH analog MR-409, although promoting tumor cell growth in vitro, exerts antitumor effects in different experimental human cancers in vivo, through the downregulation of pituitary GHRH-R and SV1 in the pituitary gland and tumors, suggesting that, as in the case of the antagonists, the reduction of GHRH-Rs leads to inhibition of tumor growth [50].

Despite exerting strong antitumor activity in a variety of human experimental cancers and possessing higher binding affinity for their receptors compared with earlier GHRH antagonists, MIA-602 and MIA-690 display a weak inhibitory effect on GH release [17]. Here, we observed that MIA-690 not only blunted the GHRH-induced increase of GH release in GH3-GHRHR cells, but was also able to reduce GH secretion when administered alone. Accordingly, MIA-690 completely blocked the GHRH-induced elevation of intracellular cAMP, and was even effective per se, highlighting the pivotal role of GHRH-R in the inhibitory activities of the antagonist. These findings are consistent with previous results obtained in GH3-GHRHR cells, where GHRH antagonists JV-1-36 and JV-1-38 blunted cAMP levels, although only in the presence of GHRH [23]. Of note, among the hormone-expressing pituitary cells, somatotrophs are most responsive to cAMP action. In fact, cAMP is induced by GHRH through GHRH-R-mediated activation of a G alpha stimulatory (Gαs) subunit, and has been implicated in activation of somatotroph proliferation, differentiation, and hormone secretion, as well as pathogenesis of GH-PAs [62]. Importantly, a subset of GH-PAs was found to express a mutant form of Gαs, *GNAS*, leading to an increase in adenylyl cyclase activity and cAMP levels [63]. Moreover, GH3 cells with a mutation of Gαs showed higher cAMP levels and increased proliferation rate and hormone secretion [64]. Interestingly, a dual role for somatotroph cAMP in promoting GH secretion has been recently demonstrated, while simultaneously causing DNA damage, potentially linking hormone hypersecretion to genome instability [65]. Thus, MIA antagonists may reduce GH secretion in GH3-GHRHR cells by inhibiting GHRH-R signaling and, consequently, cAMP levels, as well as downregulating the expression of GHRH-R. In contrast, we show here that MIA-690 was inactive on both basal and TRH-induced stimulation of PRL release in GH3-GHRHR cells, as well as on basal and CRH-induced secretion of ACTH in AtT20 cells.

Finally, this study demonstrates that MIA-602 and MIA-690, while being inactive in NFPA cells, reduced cell viability in human primary GH- and ACTH-PA cell cultures, although we could analyze only one ACTH-PA because of the low incidence of this tumor subtype. The inhibition was greater for MIA-690 in somatotrophs, where the combination with octreotide further reduced cell viability compared with octreotide alone. Furthermore, the mRNA for GHRH-R was strongly expressed in GH-PA cells, while SV1 levels were lower, in line with previous findings [22]. Interestingly, although only one analysis was performed, we observed high expression levels of GHRH-R mRNA in ACTH-PA cells, where lower levels of SV1 were also detected.

Furthermore, the expression levels of GHRH, SV1, and GHRH-R mRNA in NFPA cells were much lower than in GH- and ACTH-PA cells, which could partly explain the lack of effect observed with GHRH antagonists in NFPA cells.

Previous studies demonstrated the ability of earlier GHRH antagonists, MZ-4-71 and JV-1-36, to inhibit GH secretion induced by GHRH in somatotroph adenomas [22], an experiment not performed here because of the paucity of tumor samples. However, based on our results in GH3-GHRHR cells, we hypothesize a similar inhibitory effect for MIA antagonists in primary GH-secreting PA cells, although additional work is needed to clarify this important aspect.

Taken together, our in vitro results reveal a greatly augmented antitumor role for GHRH antagonists in GH- and ACTH-secreting PAs. In fact, the previous studies were mainly focused on the ability of the antagonists to reduce GH secretion, without considering other aspects, such as cell viability, cell growth, or apoptosis. Noteworthy, this is also the first demonstration of the inhibitory activities of GHRH antagonists in corticotroph adenoma cells. Therefore, along with the established potent antitumor role of these compounds in different tumors, GHRH antagonists of MIA series could represent a novel treatment option for PAs because of their ability to reduce cell viability and increase apoptosis in GH-PAs and ACTH-PAs, as well as for their inhibitory effects on GH secretion and cAMP signaling in somatotroph cell lines. Our findings also suggest that GHRH antagonists could be used in the clinic, in combination with classical pharmacological treatments, to improve their efficacy and reduce side effects.

## 5. Conclusions

In conclusion, MIA-602 and MIA-690 inhibit the growth of both GH- and ACTH-secreting PA cell lines and primary PA cells. In addition, MIA-602 and MIA-690 act synergistically with TMZ to reduce cell survival in PA cell lines. The mechanisms involved in these activities include the suppression of oncogenic pathways and activation of apoptotic signaling. In GH-secreting PA cell lines, GHRH antagonists also blunt the secretion of GH and reduce the levels of intracellular cAMP. These findings suggest that GHRH antagonists may represent novel therapeutic options for PAs, likely in combination with standard pharmacological treatments.

## Data Availability

No data to be reported.

## Supplementary Materials

The following are available online at https://www.mdpi.com/article/10.3390/cancers13163950/s1, Figure S1: Inhibitory effects of GHRH antagonists in combination with somatostatin analogs in GH3-GHRH and AtT20 cells, Figure S2: Effect of MIA-690 on PRL and ACTH secretion in PA cell lines, Figure S3: Effects of GHRH antagonists in NFPAs, Figure S4: Uncropped Figure 1B, Figure S5: Uncropped Figure 2D, Figure S6: Uncropped Figure 2E, Figure S7: Uncropped Figure 2I, Figure S8: Uncropped Figure 2J, Figure S9: Uncropped Figure 3C, Figure S10: Uncropped Figure 3D, Figure S11: Uncropped Figure 3E, Figure S12: Uncropped Figure 3H, Figure S13: Uncropped Figure 3I, Figure S14: Uncropped Figure 3J, Figure S15: Uncropped Figure 5A, Figure S16: Uncropped Figure 5B.
