# Supplementary material for: Antagonists of Growth Hormone-Releasing Hormone Inhibit the Growth of Pituitary Adenoma Cells by Hampering Oncogenic Pathways and Promoting Apoptotic Signaling
Source: Cancers (Basel). 2021 Aug 5;13(16):3950. doi: 10.3390/cancers13163950 (PMC8393969; doi:10.3390/cancers13163950)
Supplement: Supplementary file 1 [file cancers-13-03950-s001.zip › cancers-1282317-supplementary.pdf]

cancers

MDPI

Supplementary Material: Antagonists of Growth Hormone-Releasing Hormone Inhibit the Growth of Pituitary Adenoma Cells by Hampering Oncogenic Pathways and Promoting Apoptotic Signaling

Iacopo Gesmundo, Giuseppina Granato, Antonio C. Fuentes-Fayos, Clara V. Alvarez, Carlos Dieguez, Maria Chiara Zatelli, Noemi Congiusta, Dana Banfi, Nunzia Prencipe, Sheila Leone, Luigi Brunetti, Justo P. Castaño, Raúl M. Luque, Renzhi Cai, Wei Sha, Ezio Ghigo, Andrew V. Schally and Riccarda Granata

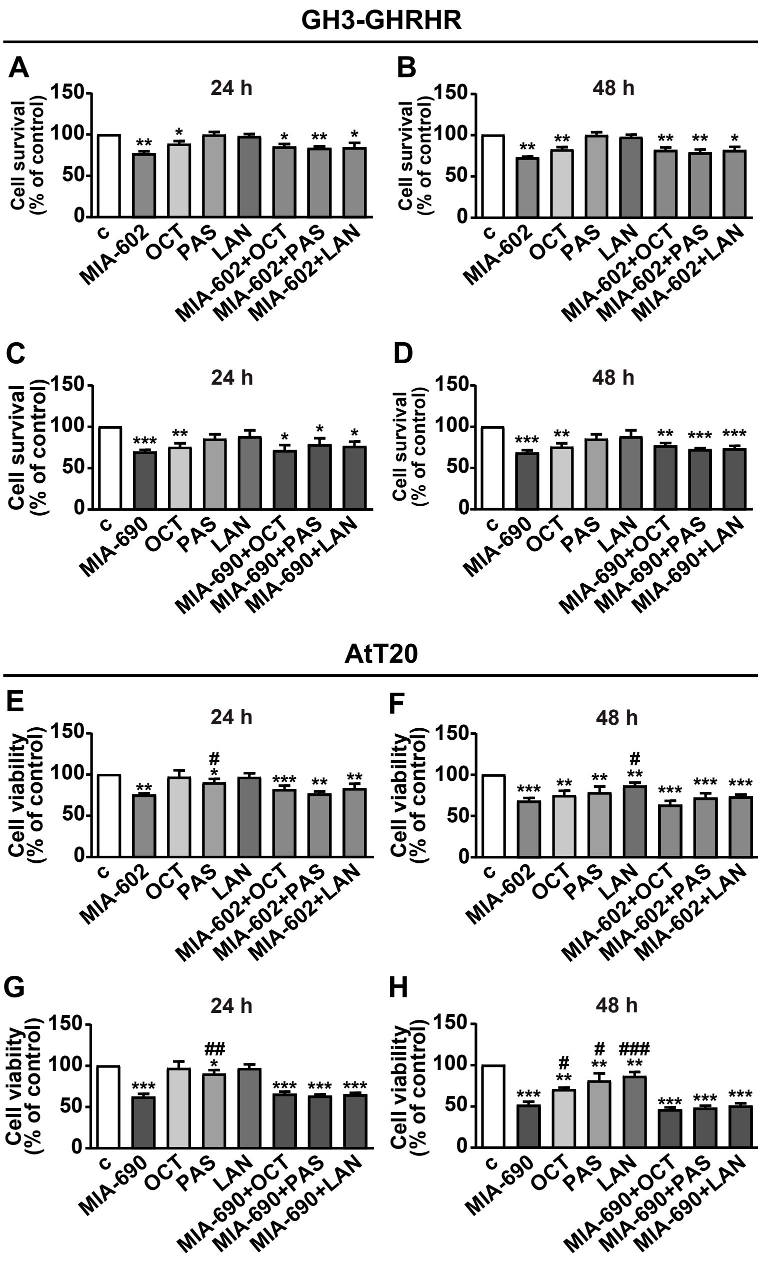

Figure S1. Inhibitory effects of GHRH antagonists in combination with somatostatin analogs in GH3-GHRH and AtT20 cells. Cell viability assessed by MTT in GH3-GHRH cells cultured in serum-deprived medium (c, control) for 24 h or 48 h with 1 μM MIA-602 **(A and B)** or 1 μM MIA-690

(C and D), and with either 0.1 μM octreotide (OCT), 0.1 μM pasireotide (PAS) and 0.1 μM lanreotide (LAN). Results, expressed as percent of control are means ± SEM of three replicates. *$p < 0.05$, **$p<$ 0.01 and **$p < 0.001$ vs. c by unpaired Student's t-test ($n = 3$). Cell viability assessed by MTT in AtT20 cells cultured in serum-deprived medium (c, control) for 24 h or 48 h with 1 μM MIA-602 **(E and F)** or 1 μM MIA-690 **(G and H)**, and with either 0.1 μM octreotide (OCT), 0.1 μM pasireotide (PAS) and 0.1 μM lanreotide (LAN). Results, expressed as percent of control are means ± SEM of three replicates. *$p < 0.05$, **$p < 0.01$ and ***$p< 0.001$ vs. c; $^{\#}p < 0.05$, $^{\#\#}p < 0.01$ and $^{\#\#\#}p < 0.001$ vs. MIA-602 or MIA-690 by unpaired Student's t-test ($n = 3$).

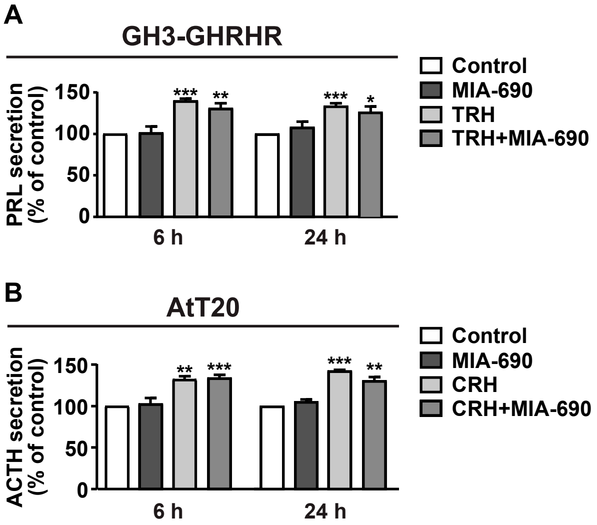

Figure S2. Effect of MIA-690 on PRL and ACTH secretion in PA cell lines. GH3-GHRHR **(A)** and AtT20 cells **(B)** were cultured in medium with 1% serum (c, control) and treated with 0.1 μM CRH or 0.1 μM TRH, respectively, in either absence or presence of 0.1 μM MIA-690 for the times indicated. Secretion of PRL and ACTH was measured by ELISA. Results, expressed as percent of control, are means ± SEM. *$p < 0.05$, **$p < 0.01$, ***$p < 0.001$ vs. c by unpaired Student's t-test ($n = 3$).

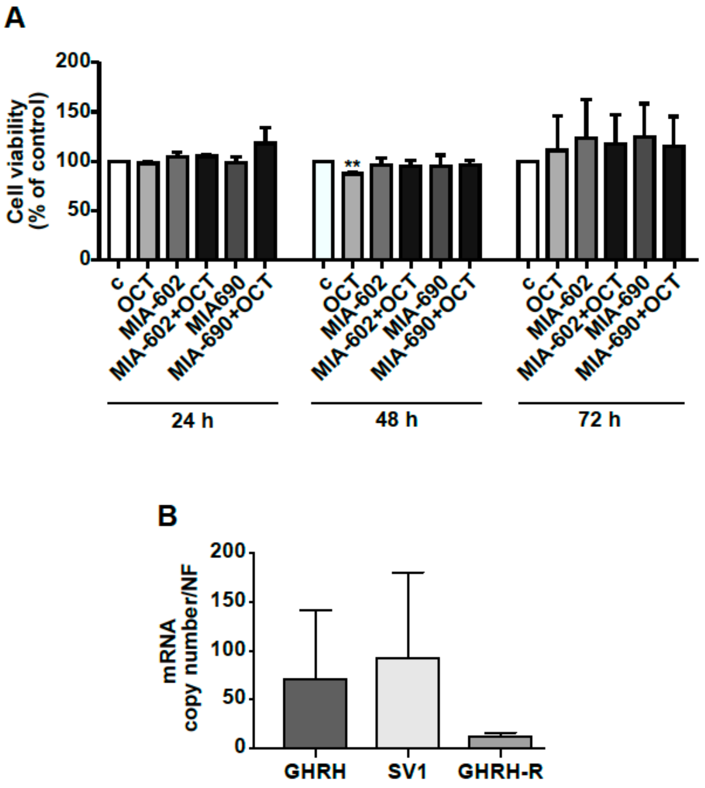

Figure S3. Effects of GHRH antagonists in NFPAs. (A) Cell viability, assessed by Alamar blue assay, in human primary cells derived from NFPAs. The cells were either untreated (c, control) or treated with 1 µM MIA-602 or 1 µM MIA-690, both alone or in combination with octreotide (OCT) for the times indicated. Results, expressed as percent of control, are means ± SEM of three replicates. **$p < 0.01$ vs. c by unpaired Student's t-test ($n = 3$). **(B)** Expression profile of GHRH, SV1 and GHRH-R in NFPAs cell cultures assessed by real-time PCR. Results represent absolute mRNA copy number adjusted by a normalization factor (NF), calculated from the expression levels of three control genes (GAPDH, ACTB and HPRT) and are means ± SEM ($n = 2$).

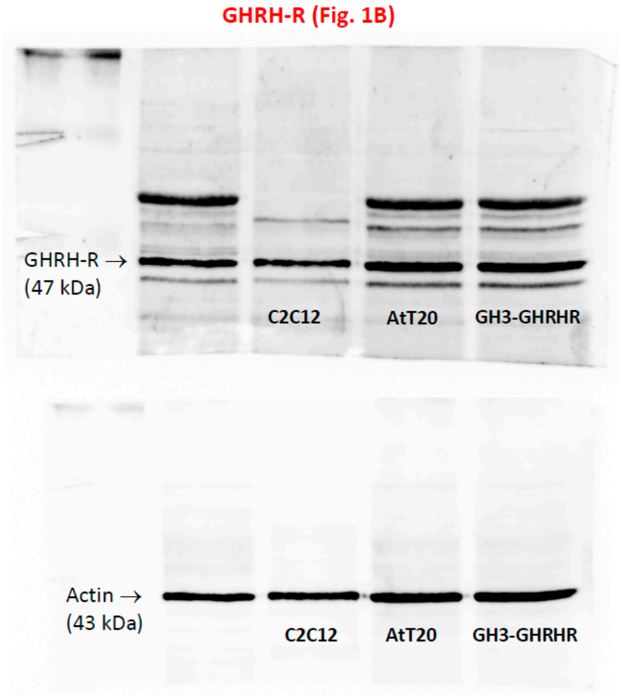

Figure S4. Uncropped Figure 1B.

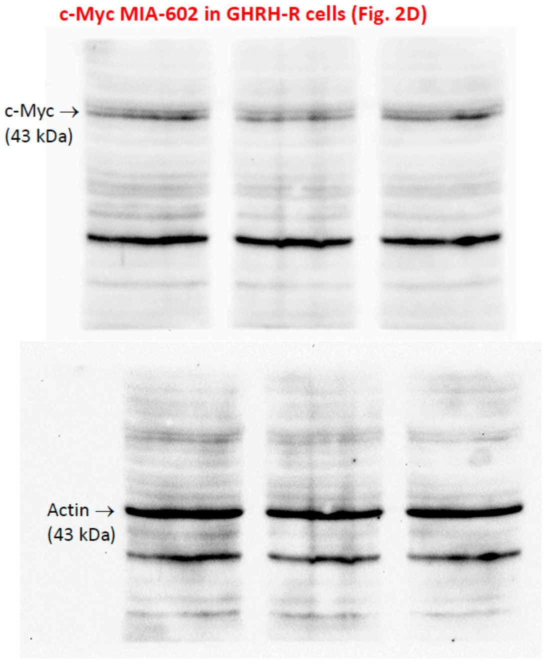

Figure S5. Uncropped Figure 2D.

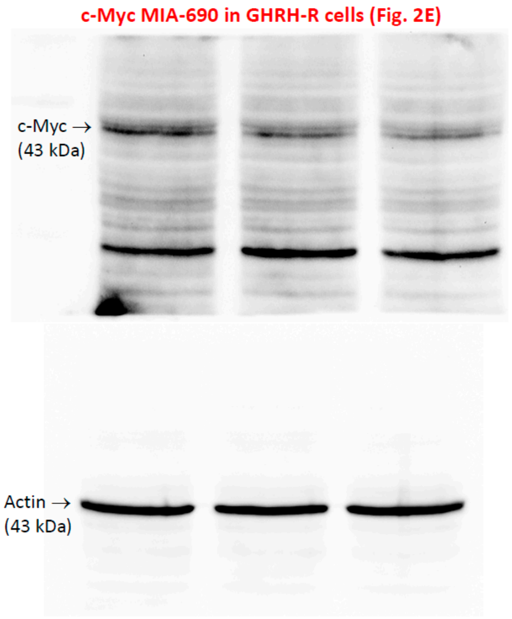

Figure S6. Uncropped Figure 2E.

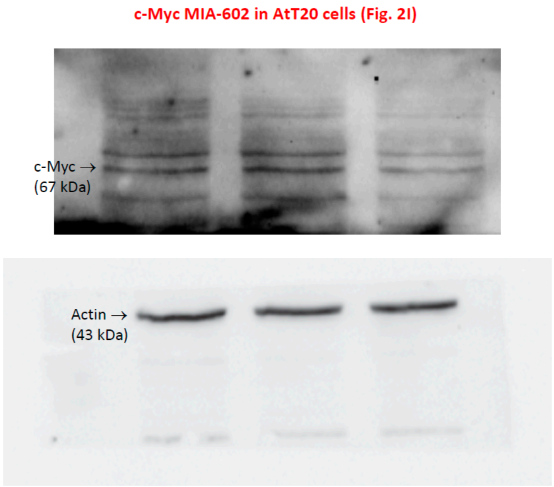

Figure S7. Uncropped Figure 2I.

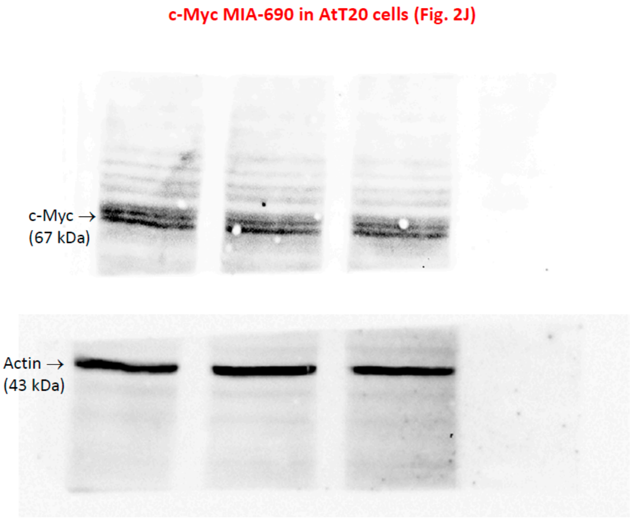

Figure S8. Uncropped Figure 2J.

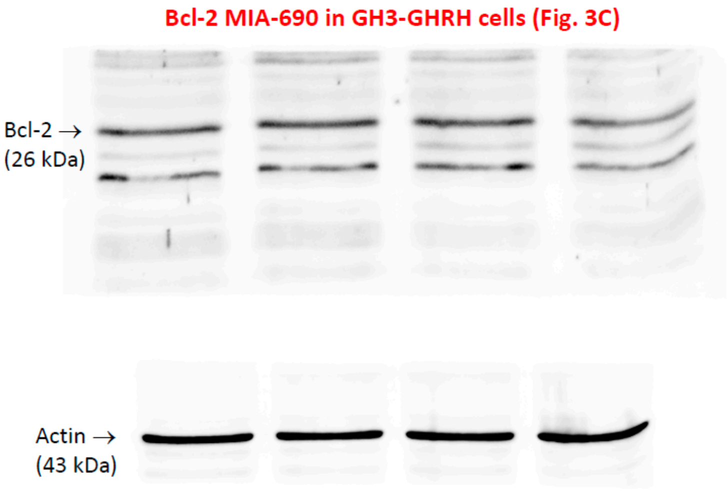

Figure S9. Uncropped Figure 3C.

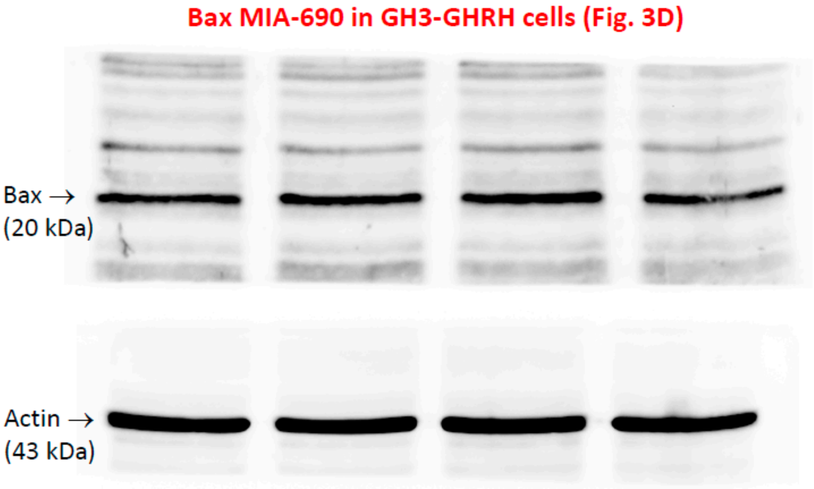

Figure S10. Uncropped Figure 3D.

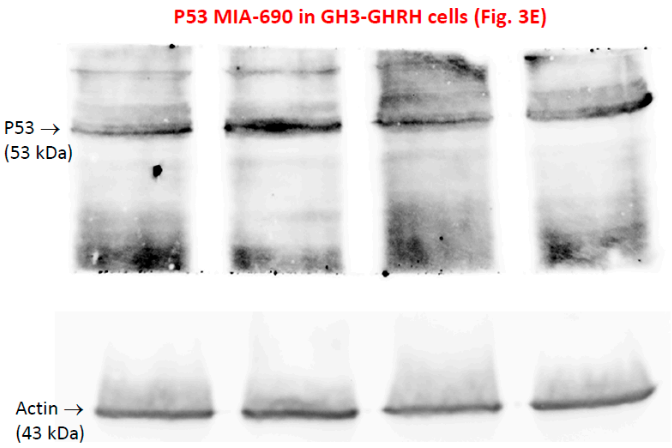

Figure S11. Uncropped Figure 3E.

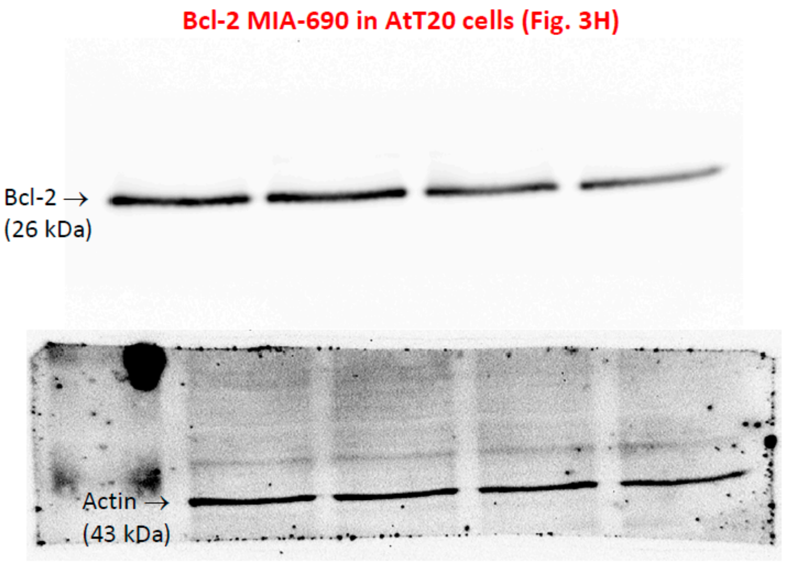

Figure S12. Uncropped Figure 3H.

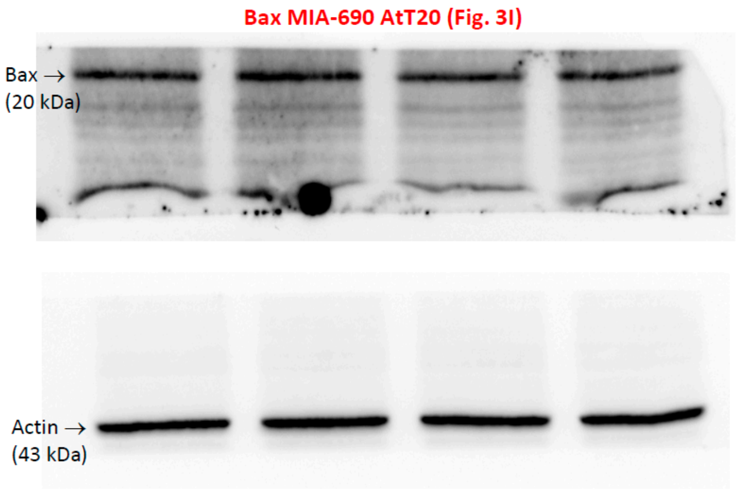

Figure S13. Uncropped Figure 3I.

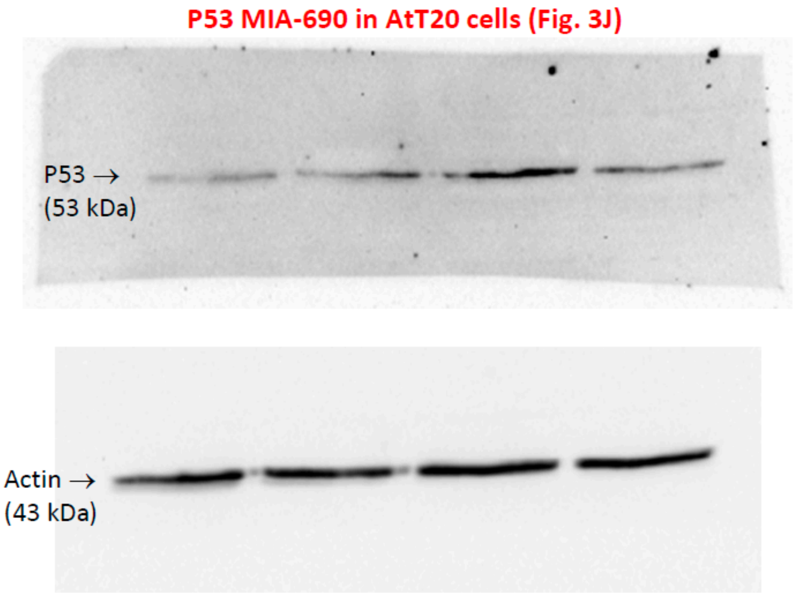

Figure S14. Uncropped Figure 3J.

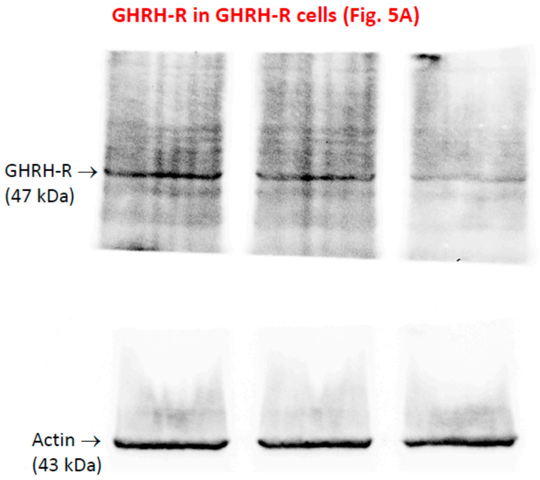

Figure S15. Uncropped Figure 5A.

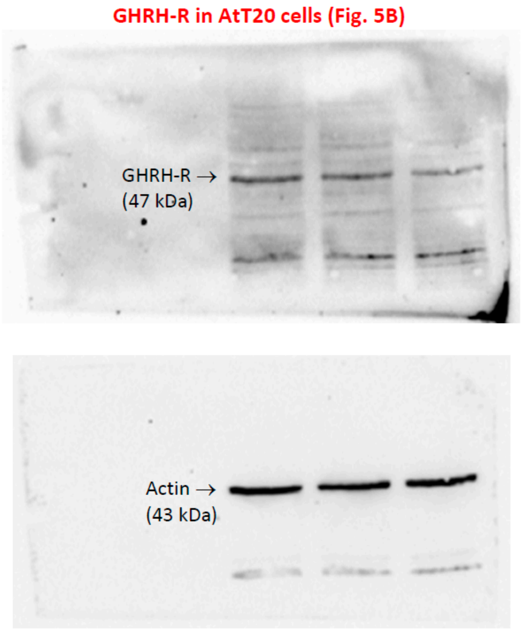

Figure S16. Uncropped Figure 5B.
